# The Changing Treatment Paradigm for Prolactinoma—A Prospective Series of 100 Consecutive Neurosurgical Cases

**DOI:** 10.1210/clinem/dgae652

**Published:** 2024-09-18

**Authors:** Victoria R van Trigt, Leontine E H Bakker, Iris C M Pelsma, Ingrid M Zandbergen, Maaia M Jentus, Mark C Kruit, Olaf M Dekkers, Wouter R van Furth, Marco J T Verstegen, Nienke R Biermasz, Dutch Prolactinoma Study Group, M E van den Akker-van Marle, M van Andel, C D Andela, C K A van den Berge, I I L Berk-Planken, P H L T Bisschop, M B Bizino, A C van Bon, J Boogaarts, C L Boot, A H Bootsma, B Burhani, S le Cessie, M L Drent, R A Feelders, E Fredriks, M Goddrie, J P de Graaf, H R Haak, J Hoogmoed, W B van den Hout, S Johannsson-Vidarsdóttir, K K Kapiteijn, M M van der Klauw, M Kramer, J M A Kuijlen, E T Massolt, J Morreau, A M Pereira Arias, W C Peul, E L Leijtens, D J Lobatto, L M Pereira Arias-Bouda, S R Ramautar, N E T Rikken, A Ritman, M A Schroijen, S Simsek, M A Sleddering, E Smolders, A M E Stades, A van der Steen, M E Stegenga, D J Stenvers, S Swinnen, S W van Thiel, M A F Traas, A C van de Ven, R A Vergeer, M Vermeulen, T M Vriesendorp, I M E Wentholt, H M de Wit, I M M J Wakelkamp, D Zagers, A H Zamanipoor Najafabadi, M S Zuurmond

**Affiliations:** Department of Medicine, Division of Endocrinology, and Center for Endocrine Tumors Leiden, Leiden University Medical Center, Albinusdreef 2, 2333 ZA Leiden, The Netherlands; Department of Medicine, Division of Endocrinology, and Center for Endocrine Tumors Leiden, Leiden University Medical Center, Albinusdreef 2, 2333 ZA Leiden, The Netherlands; Department of Medicine, Division of Endocrinology, and Center for Endocrine Tumors Leiden, Leiden University Medical Center, Albinusdreef 2, 2333 ZA Leiden, The Netherlands; Department of Medicine, Division of Endocrinology, and Center for Endocrine Tumors Leiden, Leiden University Medical Center, Albinusdreef 2, 2333 ZA Leiden, The Netherlands; Department of Pathology, Leiden University Medical Center, Albinusdreef 2, 2333 ZA Leiden, The Netherlands; Department of Radiology, Leiden University Medical Center, Albinusdreef 2, 2333 ZA Leiden, The Netherlands; Department of Medicine, Division of Endocrinology, and Center for Endocrine Tumors Leiden, Leiden University Medical Center, Albinusdreef 2, 2333 ZA Leiden, The Netherlands; Department of Neurosurgery, Leiden University Medical Center, University Neurosurgical Center Holland, Albinusdreef 2, 2333 ZA Leiden, The Netherlands; Department of Neurosurgery, Leiden University Medical Center, University Neurosurgical Center Holland, Albinusdreef 2, 2333 ZA Leiden, The Netherlands; Department of Medicine, Division of Endocrinology, and Center for Endocrine Tumors Leiden, Leiden University Medical Center, Albinusdreef 2, 2333 ZA Leiden, The Netherlands

**Keywords:** prolactinoma, transsphenoidal surgery, health-related quality of life, remission, complications

## Abstract

**Purpose:**

To evaluate patients with prolactinoma treated surgically in a time when elective prolactinoma surgery became routine in our center, using a comprehensive outcome set, focusing on preoperative assessments, surgical outcomes, and health-related quality of life (HR-QoL).

**Methods:**

Cohort of consecutive patients with prolactinoma undergoing surgery between January 2021 and August 2023. Clinical data were collected during multidisciplinary team meetings/from medical records at distinct timepoints: (1) presurgery, (2) 2 weeks postsurgery, (3) 6 months postsurgery, and (4) follow-up (median, 15.0 [10.0-24.8 months]). HR-QoL was measured using the Leiden Bothers and Needs Pituitary questionnaire. Data were described for all patients, and patients undergoing elective total resection, with additional subgroups of (1) patients undergoing a high-probability first total resection and (2) reoperations aiming for total resection.

**Results:**

One hundred surgically treated patients with prolactinoma were included (72 female). Dopamine agonist intolerance was the most frequent indication (n = 68). The surgical goal (debulking/total resection) was achieved in 90% of patients. Long-term complications occurred in 4% of patients. Seventy-eight patients underwent an elective total resection, achieving remission in 91%. The subsets of preoperatively estimated high-probability-first total resections (n = 52) and reoperations (n = 9) achieved remission in 92% and 89%, respectively. Leiden Bothers and Needs Pituitary Total Bothers and Total Needs scores improved significantly after surgery (*P* < .001, Δ-3.4 [interquartile range, −14.4 to −0.9] and *P* = .006, Δ-1.8 [interquartile range, −11.9 to 1.3]), respectively.

**Conclusion:**

High remission rates were achieved, improving HR-QoL, demonstrating (repeat) prolactinoma surgery is effective in an experienced pituitary center, as highlighted in the most recent guideline (2023).

Prolactinoma treatment aims for normalization of prolactin levels and gonadal function, with tumor shrinkage. Dopamine agonists (DAs) are the mainstay of treatment, normalizing prolactin levels in 81% of cases (1), with cabergoline being the drug of choice because of its efficacy, tolerability, and long half-life (2). However, unsatisfactory remission rates after DA withdrawal (pooled proportion: 21%) necessitate life-long treatment in most patients (1-3). Moreover, side effects, including gastrointestinal symptoms, orthostatic hypotension, and mood disturbances, are more prevalent than previously considered (1, 4).

Transsphenoidal surgery (TSS) has long been a last-resort treatment for patients suffering from severe DA intolerance or resistance. However, retrospective studies reported early surgical remission rates of 80% to 100% in microadenomas, and long-term complication rates of approximately 5%, with beneficial cost-efficiency compared to DAs (1, 5-11). These retrospective data led to reappraisal of prolactinoma treatment, with surgery being considered as a potential first-line treatment—when performed in a center of expertise—for microadenomas and well-circumscribed macroadenomas in the most recent guideline (2).

With this game-changing concept, new challenges, and perspectives on prolactinoma care arose, highlighting the importance of personalized shared decision-making weighing surgical probabilities and risks. Moreover, because DA treatment is effective and safe, surgical results must be excellent to be considered a viable alternative. Therefore, knowledge of surgical outcomes, including clinician-reported and patient-reported outcome measures, is essential.

For prolactinoma, prospective surgical data, and data on health-related quality of life (HR-QoL) is scarce. Furthermore, prior studies often lack detailed descriptions of patient subgroups based on surgical indications and preoperative estimations of total resection, hampering in-depth preoperative counseling. Because our center was at the frontline of the transition from solely DA treatment to offering both DA treatment and surgery, partially resulting from the PRolaCT study (12), the current study reported on the evaluation of our prospective surgical cohort using a comprehensive outcome set relevant for surgical decision making, focusing on surgical outcomes and HR-QoL. This analysis also included subgroup descriptions clinically relevant for counseling (based on surgical goals, probability of total resection, and reoperations).

## Methods

### Patient Population and Study Setting

This study included patients with prolactinoma, aged >18 years, treated at the outpatient clinic of the Leiden University Medical Center (LUMC), a tertiary referral center for pituitary care and (inter)nationally endorsed center of expertise within the European Reference Network on Rare Endocrine Conditions (13). Although surgeries for prolactinoma were increasingly offered as an (early) alternative to medical treatment in our expert center, the majority of patients had been treated (shortly) with DAs before referral. Prolactinoma diagnosis was based on the combination of symptomatic hyperprolactinemia (eg, galactorrhea, signs of hypogonadism), a radiologically confirmed pituitary mass on conventional magnetic resonance imaging (MRI) or functional imaging, and exclusion of other causes of hyperprolactinemia (ie, medication or nonfunctioning adenomas with mild hyperprolactinemia resulting from stalk compression). The need for written informed consent was waived by the Scientific Committee (research protocol W2018.048).

**Figure 1.**
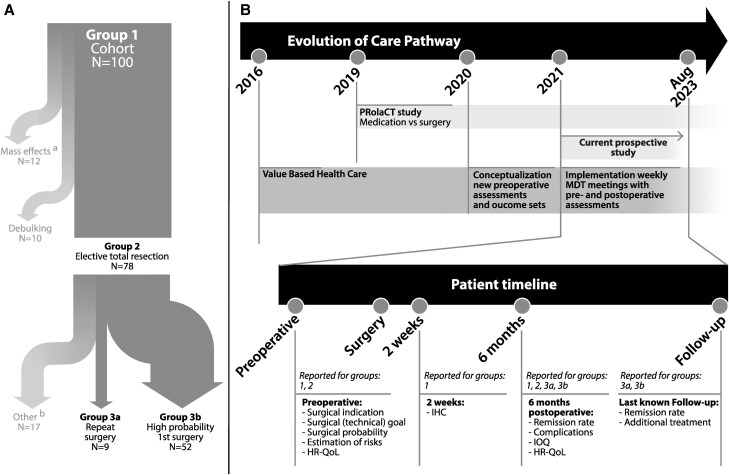
(A) Visualization of the subgroups in relation to the cohort, (B) evolution of the care pathway in time, and timepoints of data collection for the current study. The timepoints are described for the indicated (sub)groups in the main text and are available in the Supplementary Tables for the other (sub)groups. The current cohort partially overlaps with the PRolaCT study. Abbreviations: HR-QoL, Health-related quality of life; IHC, immunohistochemistry; IOQ, integrated outcome quadrants; MDT, multidisciplinary team. *^a^*Including 3 patients who underwent emergency surgery because of an apoplexy. *^b^*Patients with a lower probability of achieving total resection, yet undergoing a surgical attempt because of a high need for alternative treatment. These patients were not described separately.

Starting from 2016, a care pathway was implemented following the concepts of Value Based Health Care, and prospective data collection commenced (14). In 2019, the PRolaCT study was initiated, with elaborate prospective clinical and biochemical data collection for prolactinoma, leading to increasing numbers of referrals for prolactinoma surgery (12). In 2020, the multidisciplinary team (MDT) started conceptualizing a method of systematic pre- and postoperative assessment for all patients with pituitary adenoma using a comprehensive outcome set (15, 16). From 2021 onwards, this systematic assessment, including surgical indications and goals, estimations of probabilities and risks, and evaluation of outcomes, took place during weekly MDT meetings as part of the care pathway for all patients undergoing pituitary surgery. Figure 1 illustrates the evolution of the care pathway.

The present prospective cohort study comprised all consecutive patients undergoing TSS for a prolactinoma at the LUMC between January 2021 and August 2023. This cohort partially overlapped with the PRolaCT cohort (12), with patients undergoing an elective total resection being the primary focus of the present study. Elective total resection was defined as surgery not performed for an emergency indication (ie, apoplexy, cerebrospinal fluid leakage, or mass effects) or debulking, and was preceded by elaborate preoperative counseling. Two subgroups were studied: (1) the group undergoing a high-probability-first-time operation comprised patients harboring a micro- or macroadenoma (Knosp ≤2 (17)) undergoing elective first-time TSS without prior radiotherapy to the pituitary region, aiming for total resection with an estimated high probability of achieving this goal, and (2) the group undergoing an elective reoperation comprising of patients undergoing elective re-TSS aiming for total resection, without prior pituitary radiotherapy (Fig. 1).

### Care Pathway and Data Collection

Patients were treated according to international guidelines (2, 18), following a care pathway as described previously (19). Data were collected for standard care evaluation during weekly MDT meetings at 4 timepoints: (1) preoperatively: surgical indications, surgical goals, estimated probabilities of achieving the goals and risk of complications, and HR-QoL; (2) 2 weeks postoperatively: short-term complications and immunohistochemistry; (3) 6 months postoperatively: persisting complications, clinical and biochemical outcomes, HR-QoL; and data for (4) the follow-up (biochemical and clinical outcomes) were collected from the electronic patient records on April 11, 2024 (Fig. 1).

### Study Parameters

The following demographics were collected from the patients' medical records: age, gender, duration of disease (time between year of surgery and diagnosis), type of previous treatment (medication, TSS, radiotherapy), and duration of DA treatment (<6 months, 6 months-1 year, >1 year) when applicable. Tumor characteristics were reported as described in MRI reports by experienced neuroradiologists and reevaluated by the MDT: tumor size at diagnosis and at time of surgery (defined as not visible; microadenoma [<1 cm]; macroadenoma [1-4 cm]; giant adenoma [≥4 cm]), cavernous sinus invasion (Knosp 1-4 (17)), and optic chiasm compression (yes/uncertain/no). Pituitary neuroendocrine tumors (PitNETs) were reported and classified according to the standardized diagnostic approach to PitNETs of the European Pituitary Pathology Group (20) and World Health Organization Classification of Tumors 2022 (21, 22). The Ki-67 index was reported (≤1%, >1%-<3%, ≥3%-<10%, or ≥10%). Immunohistochemistry was considered confirmative of a prolactinoma when a localization of a PitNET of PIT1 lineage with prolactin expression was found. Other findings were considered not confirmative, yet not excluding the possibility of a prolactinoma.

### Preoperative Outcomes

The indications for surgery (ie, hormonal overproduction, mass effect, or other) were reported. Patients were considered intolerant when side effects of treatment were perceived to be inacceptable by the patient and treating physician. DA resistance was defined as persisting hyperprolactinemia, with or without tumor shrinkage, while on the maximum tolerated DA dose (≥2 mg/week of cabergoline, ≥7.5 mg/day of bromocriptine, or ≥150 mcg/day of quinagolide (19, 23)). Patients not achieving normoprolactinemia or tumor shrinkage, yet not on the doses mentioned previously because of side effects, were classified as being both intolerant and partially resistant. The group having a strong “patient preference” may have been pretreated with DA, potentially influencing the patients' treatment choices.

The primary surgical goal depended on the indication for surgery (goals for hormonal overproduction: remission/medication reduction/irradiation field reduction/symptom relief; goals for mass effects: preventive [no compression yet]/to prevent visual disturbances [with compression]/to restore visual disturbances/to restore neurological deficits; goal for “other”: to relieve headache), multiple goals could apply. The primary surgical technical goal was either total resection or debulking. The estimation of the probability to achieve the primary surgical (technical) goal was based predominantly on radiological parameters (ie, visibility, relation to cavernous sinus and/or stalk, aspects of consistency, previous imaging to assess original volume and shrinkage on DA, information of previous surgeries and the neurosurgeon's experience level) and was classified as: unlikely, possibly, or likely, as reported previously (15, 24). The estimations were prospectively recorded and used in shared decision-making weighing individual risks and possibilities. The estimated complication risk was classified as standard (∼≤5%) or elevated (∼>5%), also based on the criteria provided previously.

### Postoperative Outcomes

Surgical outcomes were reported using integrated outcome quadrants (IOQ) to combine outcomes for efficacy and safety (16). The combination between achievement of the primary surgical goal and occurrence of permanent surgical complications was considered most suitable. Outcomes were therefore categorized as: IOQ1: primary surgical goal achieved without permanent complications; IOQ2: primary surgical goal achieved with permanent complications; IOQ3: primary surgical goal not achieved without permanent complications; and IOQ4: primary surgical goal not achieved with permanent complications.

Endocrine outcomes were categorized as either biochemical remission (prolactin <1.0 × upper limit of normal [ULN]), clinical remission (prolactin <2.0 × ULN, asymptomatic, with restoration of hypogonadism, no treatment indication on clinical grounds, and no clear remnant on MRI), persistent disease (prolactin >1.0 × ULN with symptoms requiring treatment without having reached remission), or recurrence (prolactin >1.0 × ULN with symptoms requiring treatment after having reached remission). When the general term remission was used, both biochemical and clinical remissions were considered. Surgical complications were reported as transient when resolving within the follow-up period and permanent when persisting. Adverse events occurring during perioperative period, yet not induced by TSS were reported as such.

### Patient-reported Outcome Measures

HR-QoL was assessed using the Leiden Bothers and Needs Pituitary (LBNQ-P) (25)—a questionnaire assessing the disease burden of pituitary diseases, which was based on focus groups of patients with pituitary conditions, including prolactinoma. Thirty-three items cover 5 subscales: mood problems, negative illness perceptions, issues in sexual functioning, issues in social functioning, and physical and cognitive complaints. Total Bothers and Total Needs scores can be derived by addition of all Bothers and Needs subscales, respectively, divided by the number of items. The Total Bothers scores and Total Needs scores each range from 0 to 100, with higher scores indicating more pituitary-related complaints, and a higher need for professional attention for these complaints, respectively. Higher Total Bothers and Total Needs scores, therefore, indicate a higher disease burden. LBNQ-P was sent digitally preoperatively and 6 months to 1 year postoperatively, allowing sufficient recovery time (14).

### Surgical Technique

The surgical technique has been described extensively (15). In brief, TSS was performed by 2 experienced endoscopic pituitary surgeons with a 3-to-4 hands technique. Clinical experience with prolactinoma surgery dictates opening the medial compartment of the cavernous sinus in some cases because prolactinomas often have a close relationship with the cavernous sinus, particularly the medial wall, which cannot be fully anticipated based on preoperative imaging.

### Hormonal Assays

A Cobas E602 immuno-analyzer using the Elecsys Prolactin II kit of Roche Diagnostics was used to measure prolactin levels (Mannheim, Germany). Measurement range was 0.047 to 470 ng/mL (1.00-10 000 mIU/L). No high-dose hook effect occurred up to 12 690 ng/mL. Based on >400 measurements of internal quality control samples, the variation coefficient was 2.55% at 49.7 ng/mL and 2.38% at 5.9 ng/mL.

### Data Description and Analysis

IBM SPSS statistics 29 (IBM Corp. Armonk, NY, USA) was used for data descriptions. Data were reported as median (interquartile range) for continuous variables, and frequency (percentage) for dichotomous variables.

The difference between preoperative and postoperative Total Bothers and Total Needs scores were analyzed for the cohort using a Wilcoxon signed-rank test, including only patients with complete pre- and postoperative LBNQ-P data (n = 48). Statistical testing was not performed on the subgroups because these were small. *P* < .05 was considered statistically significant.

## Results

### Full Cohort (n = 100)

#### Preoperative assessment

##### Patient and tumor characteristics

The cohort consisted of 100 patients (72 females) with a median age of 35.0 (28.0-44.3) years, and median disease duration of 3 (1-6) years at the time of surgery. Patient demographics and tumor characteristics are shown in Table 1, with gender-stratified data in Supplementary Tables S1 to S6 (26). At the time of surgery, a microadenoma, macroadenoma, or giant adenoma was visible in 52, 40, and 5 patients, respectively. No clear adenoma was visible in 3 patients. Sixteen patients had Knosp scores >2, and the optic chiasm was compressed in 13 patients. Ten patients underwent functional imaging before TSS to improve preoperative assessment (27), including all patients without a visible lesion on conventional MRI. Mild growth hormone (GH) co-secretion without signs of acromegaly was present in 9 patients. In total, 96 patients underwent pharmacological pretreatment (of whom 63 patients for >1 year). Fourteen patients had undergone prior surgery (1 prior surgery, n = 12; 2 prior surgeries, n = 2). One male had undergone prior pituitary radiotherapy.

**Table 1.** Demographics and tumor characteristics at time of surgery

Parameters		Cohort N = 100	Elective total resection N = 78	High-probability first total resection N = 52	Reoperation for total resection N = 9
Age, y		35.0 (28.0-44.3)	33.5 (27.8-42.0)	32.5 (27.0-41.0)	30.0 (24.5-46.0)
Gender	(female)	72 (72.0%)	61 (78.2%)	40 (76.9%)	7 (77.8%)
Disease duration	(y)	3 (1-6)	3 (1-6)	3 (1-5)	5 (4-8)
Prolactin levels	At diagnosis (×ULN)*^a^*	7.3 (2.7-31.7)	4.6 (2.5-9.2)	3.5 (2.2-12.3)	4.9 (2.9-48.0)
	Before surgery (×ULN)*^b^*	3.7 (1.5-9.0)	4.1 (2.3-7.4)	3.3 (1.4-6.6)	2.9 (2.1-9.5)
Pituitary failure	Uncertain*^c^*	6 (6.0%)	3 (3.8%)	3 (5.8%)	0
	Yes	11 (11.0%)	3 (3.8%)	2 (3.8%)	1 (11.1%)
	ACTH	10 (10.0%)	2 (2.6%)	1 (1.9%)	1 (11.1%)
	TSH	7 (7.0%)	2 (2.6%)	2 (3.8%)	0
	GH	2 (2.0%)	1 (1.3%)	0	0
	AVP deficiency	0	0	0	0
FSH/LH suppression		36 (36.0%)	27 (34.6%)	16 (30.8%)	4 (44.4%)
Tumor size	Not visible	3 (3.0%)	3 (3.8%)	0	1 (11.1%)
	Microadenoma	52 (52.0%)	49 (62.8%)	32 (61.5%)	6 (66.7%)
	Macroadenoma	40 (40.0%)	26 (33.3%)	20 (38.5%)	2 (22.2%)
	Giant adenoma	5 (5.0%)	0	0	0
Relation to CS	Knosp 1	16 (16.0%)	12 (15.4%)	7 (13.5%)	3 (33.3%)
	Knosp 2	3 (3.0%)	2 (2.6%)	1 (1.9%)	0
	Knosp 3a	4 (4.0%)	1 (1.3%)	0	1 (11.1%)
	Knosp 3b	7 (7.0%)	4 (5.1%)	0	0
	Knosp 4	5 (5.0%)	0	0	0
Optic chiasm compression	Yes	13 (13.0%)	0	0	0
	Uncertain	1 (1.0%)	0	0	0
Functional imaging	Yes	10 (10.0%)	9 (11.5%)	1 (1.9%)	4 (44.4%)
Pretreatment	Yes	96 (96.0%)	75 (96.2%)	49 (94.2%)	9 (100.0%)
	Pharmacological	96 (96.0%)	75 (96.2%)	48 (92.3%)	9 (100.0%)
	DA (<6 mo)	11 (11.0%)	9 (11.5%)	5 (9.6%)	1 (11.1%)
	DA (6 mo-1 y)	17 (17.0%)	14 (17.9%)	10 (19.2%)	2 (22.2%)
	DA (>1 y)	63 (63.0%)	46 (59.0%)	31 (59.6%)	6 (66.7%)
	Duration unknown	5 (5.0%)	5 (6.4%)	3 (5.8%)	0
	Somatostatin analogue	2 (2.0%)	0	0	0
	GH-receptor antagonist	1 (1.0%)	0	0	0
	Surgery	14 (14.0%)*^d^*	9 (11.5%)	0	9 (100.0%)
	Radiotherapy	1 (1.0%)	0	0	0

Demographics and tumor characteristics at time of surgery for the cohort and the subgroups separately. Data are presented as median (IQR) or number (%).

Abbreviations: ACTH, adrenocorticotropic hormone; AVP, arginine vasopressin; CS, cavernous sinus; DA, dopamine agonist; GH, growth hormone; TSH, thyroid-stimulating hormone; ×ULN, times upper limit of normal.

^*a*^Data available for 78 patients (elective total resection, n = 62; high-probability-first total resection, n = 42; reoperation for total resection, n = 7).

^*b*^23 patients were on DA treatment (elective total resection, n = 10; high-probability first total resection, n = 9; reoperation for total resection, n = 0).

^*c*^Not formally assessed.

^*d*^2 patients underwent 2 prior surgeries.

##### Surgical indications and goals

Indications for TSS were hormonal overproduction (n = 87; including DA intolerance n = 68, [partial] DA resistance n = 23, or patient preference n = 22), or mass effects (n = 12; including 3 patients with an emergency indication because of an apoplexy), and other (severe headache [n = 1]). Surgical indications and goals are described in more detail in Table 2.

**Table 2.** Preoperative assessment

	Total cohort N = 100	Elective total resection N = 78	High-probability first total resection N = 52	Reoperation for total resection N = 9
**Primary indication**				
Hormonal overproduction	87 (87.0%)	78 (100.0%)	52 (100.0%)	9 (100.0%)
DA intolerance*^a^*	68 (68.0%)	62 (79.5%)	39 (75.0%)	8 (88.9%)
(Partial) DA resistanc*e^a^*	23 (23.0%)	17 (21.8%)	13 (25.0%)	1 (11.1%)
Patient preference*^a^*	22 (22.0%)	19 (24.4%)	15 (28.8%)	2 (22.2%)
Mass effect*^b^*	12 (12.0%)	0	0	0
Compression visual system*^a^*	10 (10.0%)	0	0	0
Compression of pituitary*^a^*	2 (2.0%)	0	0	0
Cranial nerve palsy*^a^*	2 (2.0%)	0	0	0
Growth*^a^*	3 (3.0%)	0	0	0
Other: severe headache	1 (1.0%)	0	0	0
**Primary goal**—**hormonal overproduction**				
Remission*^a^*	85 (85.0%)	78 (100.0%)	52 (100.0%)	9 (100.0%)
Symptom relief*^a^*	3 (3.0%)	2 (2.6%)	0	0
Medication reduction*^a^*	7 (7.0%)	0	0	0
Irradiation field reduction*^a^*	4 (4.0%)	0	0	0
**Primary goal**—**mass effect***^b^*				
Prevent visual disturbances*^a^*	4 (4.0%)	0	0	0
Restore visual disturbances*^a^*	7 (7.0%)	0	0	0
Restore neurological deficit*^a^*	2 (2.0%)	0	0	0
Preventive (no compression yet)*^a^*	1 (1.0%)	0	0	0
**Primary goal—other**				
Relieve headache	1 (1.0%)	0	0	0
**Primary surgical technical goal**				
Total resection	88 (88.0%)	78 (100.0%)	52 (100.0%)	9 (100.0%)
Debulking	12 (12.0%)	0	0	0
**Estimation of surgical risks**				
Standard	84 (84.0%)	70 (89.7%)	48 (92.3%)	9 (100.0%)
Elevated	16 (16.0%)	6 (7.7%)	4 (7.7%)	0

Estimation of surgical probability (total resection)*^c^*	N = 88	N = 78	N = 52	N = 9
Unlikely	4 (4.5%)	4 (5.1%)	0	2 (22.2%)
Potentially	23 (26.1%)	21 (26.9%)	0	6 (66.7%)
Likely	61 (69.3%)	53 (67.9%)	52 (100.0%)	1 (11.1%)

Preoperative assessment as registered preoperatively for the cohort and the subgroups separately. Data are presented as number (%).

Abbreviation: DA, dopamine agonist.

^*a*^Multiple indications may apply.

^*b*^Three patients underwent emergency surgery because of an apoplexy.

^*c*^Data shown for patients undergoing surgery aiming for total resection.

For patients undergoing surgery because of hormonal overproduction, the primary goals were disease remission in 85/87 of patients, to relieve prolactinoma-related symptoms in 3/87 patients, to reduce medication in 7/87 patients, and/or to reduce the irradiation field in 4/87 patients. For patients with mass effects, the primary goal of surgery was preventive (no compression yet) in 1/12 patients (patient with DA resistance), to prevent visual disturbances in 4/12 patients already having chiasmal compression on preoperative imaging, and to restore visual disturbances or neurological deficits in in 7/12 and 2/12 patients, respectively. In the patient with severe persisting headaches, surgery aimed to relieve the pain. The primary surgical technical goal was total resection in 88 patients, and debulking for DA dose decrease in 12 patients. Surgical risks were estimated to be standard in 84 cases and elevated in 16 patients, mostly caused by increased risk of new arginine vasopressin (AVP) deficiency (n = 6) or anterior pituitary deficiencies (n = 5).

#### Surgical outcomes

##### Short-term surgical outcomes

Immunohistochemistry was confirmative of a prolactinoma in 86/100 patients, with GH co-staining in 6/86 patients. Table 3 shows an overview of immunohistochemistry results, with pathological classifying diagnoses in Supplementary Table S3b (26). The primary surgical technical goal (ie, total resection or debulking) was achieved in 90 patients (female: n = 66 [91.7%], male: n = 24 [85.7%]). Transient complications or adverse events occurred in 11 patients, consisting mostly of syndrome of inappropriate antidiuretic hormone secretion necessitating readmission (n = 3), or sinusitis necessitating antibiotics (n = 3). An overview of all transient complications is shown in Supplementary Table S4 (26). Permanent surgical complications occurred in 4 patients, consisting of partial AVP deficiencies (n = 3), and an increase of visual field defects in a patient with a giant adenoma (n = 1). No novel permanent anterior pituitary deficiencies occurred. Adverse events that were not associated with the surgical intervention were aggravation of an anxiety disorder (n = 1), aggravation of preexisting neuropathic maxillary pain (n = 1), and an unexplained small asymptomatic cerebellar infarction found on postoperative imaging (n = 1).

**Table 3.** Immunohistochemistry and outcomes at 6 months’ postsurgery

	Cohort N = 100	Elective total resection N = 78	High-probability first total resection N = 52	Reoperation for total resection N = 9
**Immunohistochemistry**				
Consistent with clinical diagnosis prolactinoma	86 (86.0%)	66 (84.6%)	47 (90.4%)	7 (77.8%)
GH costaining	6 (29.0%)	3 (85.9%)	3 (5.8%)	2 (22.2%)
Prolactinoma not confirmed*^a^*	14 (14.0%)	12 (15.4%)	5 (9.6%)	2 (22.2%)
* *Ki67 index %*^b^*				
* *≤1%	44 (51.2%)	33 (50.0%)	20 (42.6%)	4 (57.1%)
* *>1% and <3%	25 (29.0%)	21 (31.8%)	16 (34.0%)	2 (28.6%)
* *≥3% and <10%	11 (12.8%)	10 (15.2%)	10 (21.3%)	1 (14.3%)
* *≥10%	6 (7.0%)	2 (4.5%)	1 (2.1%)	0
**Surgical goal achieved**	90 (90.0%)	71 (91.0%)	48 (92.3%)	8 (88.9%)
**Biochemical remission**	67 (67.0%)	60 (76.9%)	43 (82.7%)	6 (66.7%)
**Clinical remission**	13 (13.0%)	11 (14.1%)	5 (9.6%)	2 (22.2%)
**IOQ**				
* *1	87 (87.0%)	70 (89.7%)	47 (90.4%)	8 (88.9%)
* *2	3 (3.0%)	1 (1.3%)	1 (1.9%)	0
* *3	9 (9.0%)	6 (7.7%)	3 (5.8%)	1 (11.1%)
* *4	1 (1.0%)	1 (1.3%)	1 (1.9%)	0
**Surgical complications**				
* *Transient*^c^*	11 (11.0%)	9 (11.5%)	6 (11.5%)	1 (11.1%)
* *Permanent*^c^*	4 (4.0%)	2 (2.6%)	2 (3.8%)	0
Deterioration visual field*^d^*	1 (1.0%)	0	0	0
Partial AVP deficiency	3 (3.0%)	2 (2.6%)	2 (3.8%)	0

Immunohistochemistry and outcomes at 6 months’ postsurgery for the cohort and the subgroups separately. Data are presented as number (%).

Abbreviation: IOQ, integrated outcome quadrant.

^*a*^Unsatisfactory material/hypophysitis/non-PIT1 lineage PitNET/normal pituitary tissue without tumor evidence/suggestive for hyperplasia.

^*b*^Only shown for patients with a PitNET staining positive for prolactin (cohort n = 86, elective total resection n = 66, high-probability-first total resection n = 47, reoperation for total resection n = 7).

^*c*^Number of patients with complication.

^*d*^In a patient with a giant prolactinoma.

In total, 87 (87.0%) patients reached the surgical goal without permanent complications (IOQ1), and 3 (3.0%) patients with a permanent complication (IOQ2). The primary surgical goal was not achieved without permanent complications in 9 (9.0%) patients (IOQ3), and 1 (1.0%) patient suffered a permanent complication (mild partial AVP deficiency) without achieving the primary surgical goal (IOQ4) (Table 3). An overview of changes in clinical status throughout follow-up is depicted in Supplementary Fig. S1 (26).

##### Health-related quality of life

**Figure 2.**
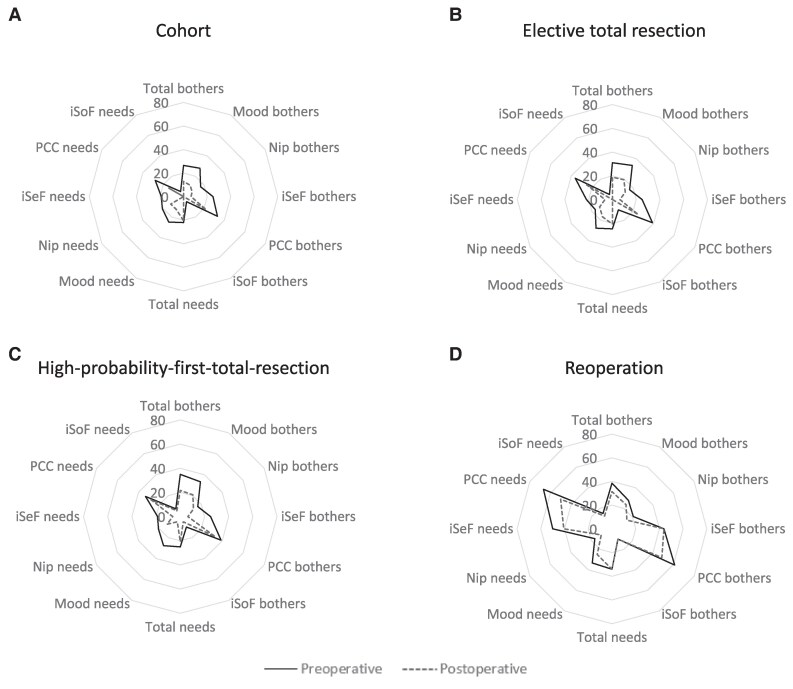
Pre- and postoperative health-related quality of life as measured by LBNQ-P, (A) for the entire cohort, n = 61 preoperative, n = 55 postoperative. Postoperative measurement median 175 (168-190) days postoperatively; (B) for patients undergoing an elective total resection, n = 50 preoperative, n = 45 postoperative. Postoperative measurement median 175 (168-192) days postoperatively; (C) for patients undergoing a high-probability first total resection, n = 35 preoperative, n = 33 postoperative. Postoperative measurement median 175 (168-189) days postoperatively; (D) for patients undergoing a reoperation for total resection, n = 5 preoperative, n = 4 postoperative. Postoperative measurement median 256 (170-341) days postoperatively. Abbreviations: iSeF, issues in sexual functioning; iSoF, issues in social functioning; LBNQ-P, Leiden Bothers and Needs Pituitary; Mood, mood problems; Nip, negative illness perceptions; PCC, physical and cognitive complaints.

LBNQ-P scores for all patients are shown in Supplementary Table S5a, with delta scores shown in Supplementary Table S5b (26). Median preoperative Total Bothers scores were 26.5 (11.0-43.2), decreasing (ie, improving) after TSS to 12.9 (2.3-31.8) (*P* < .001). Median Total Needs scores were 22.0 (9.5-43.2) preoperatively, decreasing after surgery to 14.4 (3.3-32.6) (*P* = .006). Figure 2 shows pre- and postoperative LBNQ-P scores.

For patients on DA during preoperative LBNQ-P measurement (preoperative n = 26; postoperative n = 19), median Total Bothers were 19.3 (4.9-43.2) preoperatively and 5.4 (1.5-39.4) postoperatively. Median preoperative and postoperative Total Needs were 18.9 (4.4-41.3) and 6.1 (2.3-40.9), respectively. For patients not on DA (preoperative n = 31; postoperative n = 26), median Total Bothers were 29.6 (17.4-46.2) preoperatively and 19.3 (5.7-32.4) postoperatively. Median preoperative and postoperative Total Needs were 25.8 (15.2-45.6) and 6.1 (2.3-40.9), respectively. Supplementary Fig. S2 illustrates Total Bother and Total Needs scores for prolactin levels stratified by DA treatment status (26).

For 39 patients in biochemical remission, median Total Bothers scores were 26.5 (12.1-43.2) preoperatively and 12.9 (5.3-26.5) postoperatively. Median preoperative Total Needs scores were 22.0 (8.3-42.4) and 12.1 (6.1-30.3) postoperatively.

For patients in clinical remission (data available for n = 10 preoperatively, n = 6 postoperatively), median Total Bothers scores were 21.6 (11.2-40.5) preoperatively and 3.4 (1.3-28.6) postoperatively. Median Total Needs scores were 20.8 (11.7-48.1) preoperatively and 9.9 (1.3-27.5) postoperatively.

### Elective Total Resection (n = 78)

#### Preoperative assessment

##### Estimations of surgical outcomes

The probability of achieving a total resection was estimated as likely (n = 53, 67.9%) or potentially (n = 21, 26.9%) in most patients. The probability was unlikely in 4 patients (5.1%), in whom a surgical attempt was offered as “last resort” due to severe DA intolerance or resistance, despite suboptimal surgical probabilities. The risk of complications was considered elevated in 6 patients (7.7%) (Table 2).

#### Surgical outcomes

##### Short-term surgical outcomes

Seventy-one (91.0%) patients achieved remission (biochemical remission: 60 [76.9%], clinical remission: 11 [14.1%]). Transient complications occurred in 9 (11.5%) patients, and permanent complications in 2 (2.6%) patients. Seventy (89.7%) patients achieved remission without permanent complications (IOQ1), whereas 1 (1.3%) patient achieved remission with a permanent complication (IOQ2). Six (7.7%) patients did not achieve the goal without surgical complications (IOQ3) and 1 (1.3%) patient did not achieve the surgical goal with a surgical complication (IOQ4). Clinical outcomes stratified by preoperative estimations of success are shown in Supplementary Table S3d (26).

### High-probability first total resection (n = 52)

#### Surgical outcomes

##### Short-term surgical outcomes

Remission was achieved in 48 (92.3%) patients (females n = 36 [90.0%], males n = 12 [100.0%]) with 43 (82.7%) patients in biochemical remission and 5 (9.6%) patients in clinical remission. Forty-seven (90.4%) patients achieved remission without complications (IOQ1), 1 patient (1.9%) achieved remission with a complication (IOQ2), 3 (5.8%) patients did not achieve remission without complications (IOQ3), and 1 (1.9%) patient was not in remission with a permanent complication. The permanent complications consisted of partial AVP deficiencies in 2 (3.8%) patients. Six (11.5%) patients experienced transient complications.

##### Health-related quality of life

Median Total Bothers scores were 34.9 (14.4-43.9) preoperatively, and 20.5 (6.1-33.0) postoperatively. Median Total Needs scores were 25.0 (11.4-45.5) preoperatively, and 20.5 (6.5-40.2) postoperatively.

##### Follow-up and additional treatment

Median follow-up duration of patients in biochemical remission (n = 43) was 13.8 (8.7-21.0) months. One (1.9%) patient experienced a recurrence and 1 (1.9%) went from biochemical to clinical remission.

All patients in clinical remission (n = 5) remained in clinical remission without additional treatment. Their median follow-up time was 20.8 (9.9-24.6) months.

At last known follow-up, 35 females (87.5%) and all males (n = 12, 100.0%) were in (biochemical/clinical) remission.

The 4 patients with persisting disease underwent additional treatment: repeat TSS (n = 3) and gonadal replacement therapy (n = 1).

### Reoperations (n = 9)

#### Surgical outcomes

##### Short-term surgical outcomes

Remission was achieved without permanent complications in 8 (88.9%) patients (IOQ1), whereas remission was not achieved without permanent complications in 1 (11.1%) patient (IOQ3). Biochemical and clinical remission was achieved in 6 (66.7%) and 2 (22.2%) patients, respectively. No permanent complications occurred.

##### Health-related quality of life

Median Total Bothers scores were 38.6 (14.0-43.6) preoperatively and 31.4 (4.9-50.6) postoperatively. Median Total Needs scores were 34.1 (16.7-51.5) preoperatively and 34.1 (7.6-45.8) postoperatively.

##### Follow-up and additional treatment

Median duration of follow-up was 28.8 (15.4-33.5) months (Table 4). At last known follow-up, all 8 (88.9%) patients remained in remission (biochemical remission n = 6; clinical remission n = 2). The patient with persistent disease restarted DA therapy postoperatively.

**Table 4.** Clinical outcomes at last follow-up and additional treatment

	Cohort, N = 100	Elective total resection N = 78	High-probability first total resection N = 52	Reoperation for total resection N = 9
**Duration of follow-up (mo)**	15.0 (10.0-24.8)	15.2 (10.5-24.8)	14.8 (9.0-23.2)	28.8 (15.4-33.5)
**Clinical status**				
Remission	79 (79.0%)*^a^*	71 (91.0%)*^a^*	48 (92.3%)*^a^*	8 (88.9%)
*Biochemical remission*	67 (67.0%)*^a^*	60 (76.9%)*^a^*	42 (80.8%)*^a^*	6 (66.7%)
*Clinical remission*	12 (12.0%)	11 (14.1%)	6 (11.5%)	2 (22.2%)
Persistent disease	19 (19.0%)	6 (7.7%)	3 (5.8%)	1 (11.1%)
Recurrence	1 (1.0%)	1 (1.3%)	1 (1.9%)	0
Deceased	1 (1.0%)	0	0	0
**Additional treatment**	18 (18.0%)	3 (3.8%)	2 (3.8%)	1 (11.1%)
Medication (DA)	13 (13.0%)	2 (2.6%)	0	1 (11.1%)
Surgery	3 (3.0%)	1 (1.3%)	1 (1.9%)	0
Radiotherapy	1 (1.0%)	0	0	0
Gonadal replacement therapy	1 (1.0%)	1 (1.3%)	1 (1.9%)	0

Clinical outcomes at last follow-up and details on additional treatment for the cohort and the subgroups separately. Data are presented as median (IQR) or number (%).

Abbreviation: DA, dopamine agonist.

^*a*^One patient in biochemical remission was lost to follow-up 3 months postsurgery.

### Subgroup Descriptions

Clinical and biochemical outcomes of patients undergoing a total resection stratified for duration of medical pretreatment, tumor size, invasiveness, and indication for surgery (intolerant versus resistant) were reported in Supplementary Tables S7a-d and S8a-d (26).

## Discussion

The present study described the preoperative assessment and postoperative outcomes of a consecutive cohort of patients with prolactinoma undergoing TSS. This series illustrated the new landscape of prolactinoma treatment, in which surgery was considered a potential first-line therapy for patients with noninvasive prolactinoma in our center, as the most recent guideline suggested (2). Surgery was preceded by long-term DA treatment in most patients. Generally, the surgical goal was achieved in 90% of patients. Biochemical or clinical remission was achieved in 92% of patients with noninvasive prolactinomas undergoing their first total resection, with similar remission rates in patients undergoing a reoperation. Postoperative HR-QoL improved significantly.

Our Value Based Health Care care pathway, in which surgical goals are discussed preoperatively and registered prospectively in a systematic manner, enables objective and critical analysis of surgical outcomes and improvement of treatment strategies. In the current cohort, the primary indication for surgery was DA intolerance because most patients had undergone long-term medical treatment before TSS, similar to previously described cohorts (5, 19, 28). The primary surgical technical goal—total resection for most patients—was achieved in the vast majority of patients. Similar to previous cohorts of microprolactinoma (1, 5, 6, 8, 29, 30), the subgroup analysis of patients with noninvasive prolactinoma undergoing their first surgical attempt showed high surgical remission rates (92% at 6 months follow-up, females: 90%; males: 100%), yielding important information to weigh against the outcomes on DA therapy. Notably, cabergoline was shown to induce normoprolactinemia and tumor shrinkage in 91% (95% CI, 85-96) and 88% (95% CI, 82-94) of patients with micro- and macroprolactinoma, respectively (1). Remission rates of 47% (microprolactinoma) and 41% (macroprolactinoma) have been reported for patients with considerable tumor shrinkage on low doses of cabergoline before DA withdrawal (2, 31), which was only approximately one third of all patients with prolactinoma. Yet, only 21% of patients with microadenoma and 16% of patients with macroadenoma achieve ongoing remission after DA withdrawal based on a recent meta-analysis (3, 32).

The optimal timing for surgery remains a point of discussion (19). An important contributing factor is the proposed fibrotic effect of DA treatment on the tumor, which might be detrimental to surgical success (8, 33, 34). In our cohort, no evident trend toward worse surgical outcomes with longer pretreatment was observed (Supplementary Tables S7a and S8a (26)). However, in the hands of very experienced neurosurgeons, remission and complication rates may not reflect the complexity of surgery, as outcomes remain good. Because fibrotic changes were not assessed in the current cohort and there were only few treatment-naïve patients, this aspect requires further research.

The present study also described surgical outcomes of patients with prolactinoma undergoing reoperations aiming for total resection. With prolactinoma surgery becoming a more accepted treatment modality, the question arises whether reoperations should be considered in patients not achieving remission in one attempt. Careful reappraisal of the localization of the remnant, and evaluation of the reason for initial incomplete resection is of great importance to select patients in whom total resection is feasible upon a second attempt. New imaging techniques (eg, [18F]fluoroethyl-L-tyrosine or [11C]methionine PET co-registered with magnetic resonance imaging) proved to be useful to enhance chances of success in selected cases (27, 35, 36). Moreover, our team's increasing experience with resections of the medial wall of the cavernous sinus may have led to improvement of surgical outcomes because prolactinomas are frequently localized laterally. The high remission rates (almost 90%) without permanent complications supported the notion that reoperations may be a safe and effective treatment strategy for prolactinoma in the hands of experienced neurosurgeons.

The permanent complication rate was similar to previously reported series (1, 5-11). The prospective design of our cohort enabled careful registration of surgical complications. Adverse events unlikely to be associated with surgery were reported as such to correctly represent clinical reality. A partial, mild, novel AVP deficiency was the most common permanent complication (3% in the full cohort), interestingly occurring most frequently in first surgeries of microprolactinoma. This may be explained by the localization of the adenomas, close to the posterior pituitary lobe. Fortunately, postoperative HR-QoL seemed to be comparable to patients without complication. Nevertheless, this finding stresses the importance of careful surgical indication setting and weighing of risks and benefits for each patient because not all complications can be anticipated and prevented.

Our study showed HR-QoL, as measured by the LBNQ-P, improved significantly after surgery. Interestingly, patients undergoing repeat TSS seemed to have the lowest HR-QoL, which remained most impaired after (mostly successful) surgery—although this group was small. To our knowledge, this is the first study to compare pre- and postoperative HR-QoL in a large cohort of patients with prolactinoma indicating HR-QoL data in patients with prolactinoma are scarce. In line with current findings, one of our group's prior studies describing pre- and postoperative LBNQ-P scores in pituitary patients, including 16 patients with prolactinoma, found the scores improved significantly (total LBNQ-P score 45.0 [34.4-55.6] preoperatively, and 25.9 [16-35.6] 6 months postoperatively) (14). Another study using LBNQ-P in 92 patients with well-controlled or cured prolactinoma—among whom 28% had undergone surgery—found lower (ie, better) scores (Total Bother: 10.6 [1.2-19.7], Total Needs: 11.1 [1.0-27.9]) compared to the current study (25). As described by the well-known Wilson and Cleary model, many factors impact HR-QoL, and general well-being results from a complex interplay of physiological, clinical, and social aspects (37, 38). One reason for this difference in scores could be remission duration, as patients included in the present cohort were assessed 6 months after active disease, and the earlier study included patients in longstanding remission, allowing for more recovery time. Furthermore, the present cohort might have had a higher preoperative disease burden and longer disease duration (ie, longer exposure to hormonal excess), necessitating longer recovery time. This hypothesis may also explain the persistently higher postoperative disease burden in patients not on DA preoperatively, and patients undergoing repeat TSS. Larger studies with longer postoperative follow-up are needed to scrutinize the impact of clinical and biochemical parameters on HR-QoL.

In agreement with previous findings, physical and cognitive complaints seemed to be most disabling in the present cohort, which remained most impaired after surgery (25). Physical prolactinoma-related symptoms are well known, whereas cognitive complaints remain less acknowledged. Small studies describing cognitive functioning in patients with prolactinoma found worse memory, attention, and executive functioning compared to healthy controls (39-41), which seemed to improve after surgery (42). However, these studies were small and did not adjust for relevant clinical parameters. Future studies describing more patient-reported outcomes, including a comparison to medical treatment, and larger studies focusing on cognitive functions in patients with prolactinoma are warranted.

An ongoing dilemma concerns the classification and clinical implications of patients with persistently marginally elevated prolactin levels after surgery, without symptoms or radiological tumor remnants. In our study, these patients seemed to have an increased risk of recurrence of functional hyperprolactinemia compared to patients in biochemical remission, yet 85% of patients had a satisfactory outcome more than 1 year postsurgery, remaining in clinical remission or even achieving biochemical remission without additional treatment (6.7%), with LBNQ-P scores similar to patients in biochemical remission. Persistent mild hyperprolactinemia has ample causes, including the presence of a remnant, and physiological elevations from a higher setpoint, stress, medication, exercise, high-protein meals, or alcohol consumption (2). Differentiation between physiological causes and the presence of a small remnant can be complex, especially with unspecific symptoms (eg, headache, mood disturbances). Because these patients have no indication for further treatment, albeit with a risk of a small remnant, we propose the term “clinical remission” for this group. Nonetheless, in the presence of unspecific symptoms, the possibility of persisting disease should be explored.

A few limiting aspects of the present study should be taken into consideration. First, although postoperative follow-up was relatively long compared to previous studies, a more extensive follow-up period would give more information on recurrence rates and the natural course of prolactin levels in patients in clinical remission. Second, because of careful selection of patients being eligible for reoperation, only a small subgroup of patients underwent repeat surgery aiming for total resection. Therefore, larger cohorts with more in-depth analysis of outcomes are required. Third, only approximately 60% of patients completed the pre- and postoperative HR-QoL questionnaires, possibly leading to bias. A more elaborate analysis of patient-reported outcomes will follow in the PRolaCT study (12). Last, as prolactinoma are frequently located laterally, use of a prognostic classification based on invasiveness and proliferation markers (eg, The French Five-Tiered Prognostic Classification (43)) may be relevant for future studies, albeit beyond the scope of this manuscript.

The present study emphasizes the importance of multidisciplinary preoperative assessment of indications, goals, possibilities, and risks, as they shape preoperative counseling and determine the definition of surgical success. High remission rates in patients undergoing their first total resection for prolactinoma and those undergoing repeat surgeries were observed, leading to improvement of HR-QoL, evidencing (repeat) prolactinoma surgery is a safe and effective treatment in the hands of an experienced pituitary team.

## Data Availability

The dataset analyzed during the current study is not publicly available but is available from the corresponding author on reasonable request.

## Abbreviations

AVP: arginine vasopressin
DA: dopamine agonist
HR-QoL: health-related quality of life
IOQ: integrated outcome quadrant
LBNQ-P: Leiden Bothers and Needs Pituitary
LUMC: Leiden University Medical Center
MDT: multidisciplinary team
MRI: magnetic resonance imaging
PitNET: pituitary neuroendocrine tumor
TSS: transsphenoidal surgery
ULN: upper limit of normal
